# Fracture Load of CAD/CAM Feldspathic Crowns Influenced by Abutment Material

**DOI:** 10.3390/ma13153407

**Published:** 2020-08-02

**Authors:** Mladen Bencun, Andreas Ender, Daniel B. Wiedemeier, Albert Mehl

**Affiliations:** 1 Division of Computerized Restorative Dentistry, Clinic of Conservative and Preventive Dentistry, Center of Dental Medicine, University of Zurich, Plattenstrasse 11, CH-8032 Zurich, Switzerland; bencunm@gmail.com (M.B.); albert.mehl@zzm.uzh.ch (A.M.); 2 Statistical Services, Center of Dental Medicine, University of Zurich, Plattenstrasse 11, CH-8032 Zurich, Switzerland; daniel.wiedemeier@zzm.uzh.ch

**Keywords:** CAD/CAM, elastic modulus, fracture load, feldspathic ceramic, dentin, hybrid ceramic, reinforced composite, polymethyl methacrylate

## Abstract

In vitro studies investigating the mechanical properties of dental reconstructions use various materials to replicate prepared teeth. However, no uniform recommendation exists as to which material is most suitable for standardized testing. The purpose of this study was to identify a material that resembles human dentin in fracture load tests. Sixteen human teeth were scanned with an intraoral scanner to obtain copies of the original crown morphology and were then prepared for crowns. Replica dies of the prepared teeth including the root morphology were fabricated with a Computer-aided design and computer-aided manufacturing (CAD/CAM) system and divided into four groups: (A) reinforced composite (RC); (B) human dentin (HD); (C) polymethyl methacrylate (PM); and (D) hybrid ceramic (HC). Sixty-four feldspar ceramic crowns were designed with the biocopy mode, fabricated with a CAD/CAM system, luted on the dies, and then with the roots embedded in polymethyl methacrylate. Care was taken to position all specimens of the same morphology identically. Thermo-mechanical load cycling was performed in a chewing simulator followed by fractural loading of the crowns. A mixed effect linear model was fitted to the data, and pairwise contrasts were estimated on the marginal means and corrected for multiple testing according to Tukey (α = 0.05). The means for fracture load (N) were 2435 N (95% CI (2162, 2709)) for hybrid ceramic, 1838 N (95% CI (1565, 2112)) for reinforced composite, 1670 N (95% CI (1396, 1943)) for human tooth and 1142 N (95% CI (868, 1415)) for polymethyl methacrylate abutment materials. Post-hoc pairwise contrasts revealed a statistically significant (*p* < 0.05) difference among all groups except for reinforced composite and human dentin (*p* = 0.76). The results indicate that the mechanical properties of abutment dies play a significant role for a possible substitution of natural teeth in in vitro studies.

## 1. Introduction

In prosthodontics, various ceramics are used for the single or multi-unit reconstruction of damaged teeth including feldspathic porcelain, glass–ceramic, zirconia, resin-infiltrated hybrid ceramic and metal–ceramic systems [1]. Despite the well-documented clinical success of all-ceramic restorations [2], no universal solution exists for every clinical case. Therefore, prosthodontics research and research of new materials continues [3]. Many in vitro studies are conducted to investigate the properties of these materials to transfer such findings to in vivo. For example, all-ceramic crowns or partial fixed dental prostheses are loaded until fracture to predict their clinical behavior [4]. However, these studies are not conducted uniformly. Human teeth [5] and various other materials have been used as abutment dies including polymethyl methacrylate [6], epoxy resin [7], high filler content resin [8] and stainless steel [9].

Studies have shown that the elastic modulus of the abutment material influences the fracture load of the restoration material. Ceramics are stiff and brittle and fracture under tensile stress, which is highest when the abutment material has a lower elastic modulus than the ceramic [10,11,12]. Additionally, fracture strength of ceramics is affected by the used cementation method, being higher with adhesive luting [13]. However, bond strength varies among different adhesive luting agents, which may affect the fracture strength of the ceramic reconstruction [14]. Differences in the preparation design of abutment dies may further impede agreement among study groups.

Although in many studies an abutment material is used, which is assumed to have an elastic modulus similar to dentin [15], no consensus exists on the correct elastic modulus. The elastic modulus of dentin has been reported as ranging from 5 to 42 GPa, depending on the method of measurement [4,16,17,18,19,20,21,22,23,24,25,26,27]. Additionally, differences between deciduous and permanent teeth and wet or dry conditions affect the measurements [17,26,28]. Furthermore, the elastic modulus decreases with proximity to the pulp [17]. Dentin’s tubular system is a complex and heterogeneous substance. The structure of dentin is different near the dentino-enamel junction in comparison to near the pulp [29]. The more mineralized peritubular dentin has been shown to exhibit a higher elastic modulus than the less mineralized intertubular dentin [16,20,25].

For these reasons, a material that resembles human dentin’s elastic modulus may enable fracture load measurements more transferable to clinical conditions.

Computer-aided design and computer-aided manufacturing (CAD/CAM) technology offers the possibility of standardized and automated processing of a broad variety of materials, such as resins [30], zirconia [31], glass ceramics including porcelains, hybrid ceramics and metal alloys [32]. A recent study attempted to design a methodology for standardized preparation of human tooth specimens using CAD/CAM technology [33]. However, an even preparation taking into account the pulp and tooth anatomy can be difficult with a CAD/CAM system. Additionally, CAD/CAM dental materials have been tested recently concerning hardness [34], bond strength [35] and wear [36], showing their mechanical behavior under ideal laboratory conditions. A CAD/CAM processible material that resembles human teeth in mechanical properties would eliminate the need for extracted teeth and their not standardized preparation for such testing. This would facilitate the reproducibility of studies allowing for investigation of reconstructions of variable thickness or dimensions on a standardized tooth abutment.

To date, no comparison has been made of abutments, which copy the original tooth morphology including the roots and which can be fabricated from various CAD/CAM processible materials. Therefore, the aim of this study was to investigate the influence of the elastic modulus of different abutment materials on the fracture load under the same conditions and to identify a material that resembles human teeth in mechanical properties most. For this reason, materials were chosen with elastic moduli of 3.2 GPa (Telio CAD; A3; Ivoclar Vivadent, Schaan, Liechtenstein) (PM), 10.3 GPa (BRILLIANT Crios; A3; Coltene AG, Altstätten, Switzerland) (RC) and 30 GPa (VITA ENAMIC; 2M2HT; Vita Zahnfabrik H. Rauter, Bad Säckingen, Germany) (HC) (values provided by the manufacturers). The null hypothesis of this study was that there is no significant difference (*p* < 0.05) between the fracture load of feldspathic ceramic crowns luted on prepared natural teeth and replica abutments fabricated from polymethyl methacrylate, reinforced composite or hybrid ceramic.

## 2. Materials and Methods

Single molar feldspathic CAD/CAM crowns luted on abutment replicas with different elastic moduli were fatigued and then loaded until fracture.

### 2.1. Preparation of Human Molars and Crown Design

**Figure 1:**
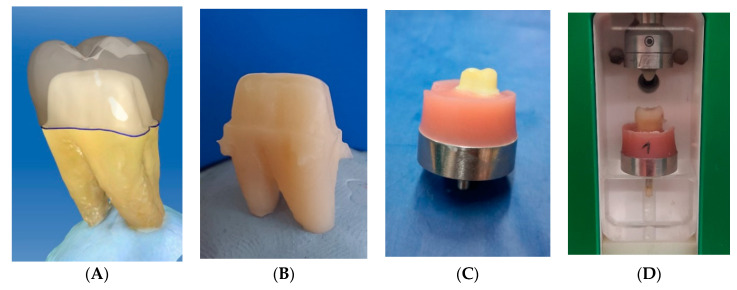
Preparation of specimens for fractural loading: (**A**) original prepared tooth with overlain biocopy crown; (**B**) hybrid ceramic (VITA ENAMIC; 2M2HT; Vita Zahnfabrik, Bad Säckingen, Germany) replica die of original preparation including root morphology; (**C**) specimen embedded in metal carrier with polymethyl methacrylate; and (**D**) thermo-mechanical load cycling in chewing simulator with human molar cusps as antagonists.

Sixteen non-carious and non-cracked mandibular molars were selected from a collection of extracted teeth of the University of Zurich (Zurich, Switzerland) and stored in 0.1% thymol solution (Sigma-Aldrich Chemie GmbH, Buchs, Switzerland) [37]. The patients had given informed consent for the anonymized use of their teeth and no approval from the local ethical committee (Kantonale Ethikkommission, Zurich, Switzerland) was required. The mandibular molars were mechanically cleaned of periodontal ligament remains and calculus using a scaler, and then polished with pumice stone and water using an occlubrush (KerrHawe SA, Bioggio, Switzerland). Afterwards, they were scanned with the Cerec Omnicam (Dentsply Sirona, York, PA, USA). These scan data were used to record the original tooth morphology for the design and fabrication of the crowns by applying the CAD mode “biocopy” (CEREC software SW 4.6 beta 2; Dentsply Sirona, York, PA, USA). After scanning, the mandibular molars were prepared for crowns following the guidelines for all-ceramic preparation: shoulder finishing line, total occlusal convergence of 10–20 degrees, occlusal reduction of at least 1 mm, radial reduction of at least 0.8 mm and minimal occlusocervical dimension of 4 mm [38]. The preparations were scanned again with the Cerec Omnicam (Dentsply Sirona, York, PA, USA) and the crowns were designed using the “biocopy” function (Figure 1A). To check the dimensions of the preparations, the parameters “minimal thickness (radial)” and “minimal thickness (occlusal)” were adjusted (radial: 800 μm; occlusal: 1000 μm). Other than that, standard design parameters for single crowns recommended by the manufacturer were used.

### 2.2. Fabrication of CAD/CAM Abutments and Restorations

A CAD/CAM milling machine (CEREC inLab MCXL milling unit, Dentsply Sirona, York, PA, USA) was used to fabricate both the replicas of the prepared teeth including the entire root morphologies (abutments) (Figure 1B) and the crowns. The crowns, designed with the biocopy mode, were made of a feldspathic ceramic (VITABLOCS Mark II; 3M2C; Vita Zahnfabrik H. Rauter, Bad Säckingen, Germany), a clinically proven material with an elastic modulus of 45 GPa, higher than the abutment materials [39]. For the abutments, each scanned sample of the prepared molars was fabricated from three different materials: polymethyl methacrylate (PM, *n* = 16) (Telio CAD; A3; Ivoclar Vivadent, Schaan, Liechtenstein), reinforced composite (RC, *n* = 16) (BRILLIANT Crios; A3; Coltene AG, Altstätten, Switzerland) and hybrid ceramic (HC, *n* = 16) (VITA ENAMIC; 2M2HT; Vita Zahnfabrik H. Rauter, Bad Säckingen, Germany) (Table 1). As control, the natural prepared teeth were used (HD, *n* = 16). In total, four groups of 16 abutment and crown specimens were made, each of which had the same root morphology, the same preparation and the same crown morphology as the respective original (in total 64 abutments and 64 crown specimens) (Figure 1A,B).

### 2.3. Embedment of Specimens

**Figure 2:**
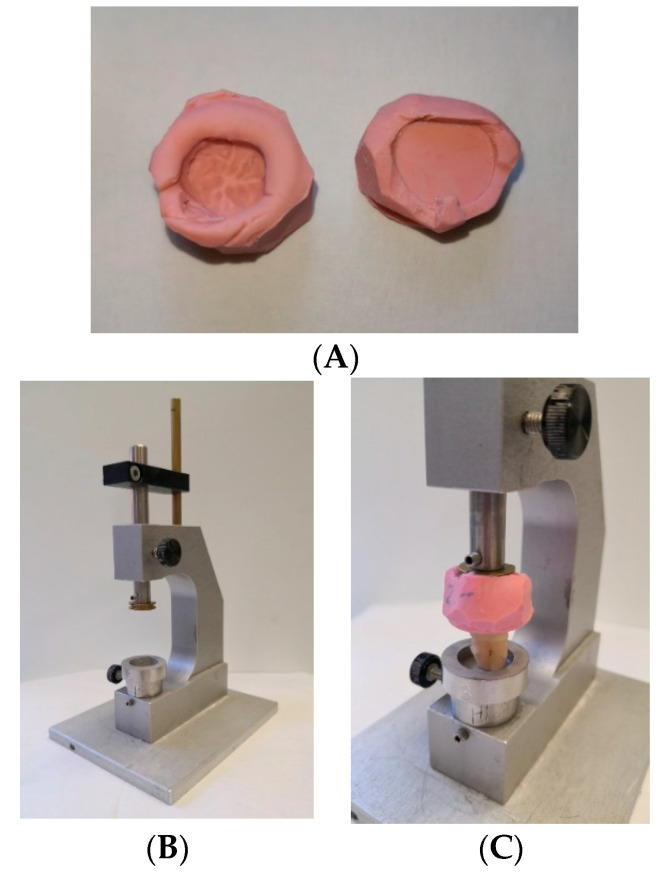
Identical positioning of specimens with same morphology. (**A**) Silicone impression: bottom side, crown of first embedded specimen; top side, asymmetrical disc. (**B**) Self-made device allowing for identical repositioning of further specimens. Vertical pin with mounted asymmetrical disc was locked so as to only allow vertical movements and no rotation. (**C**) Crown fitting into bottom side, asymmetrical disc fitting into top side of silicone impression, allowing for exact reproduction of specimen positions.

The root tips of the human teeth were ground using a grinding wheel to achieve a plane surface and allow for stable positioning. By this means, a specimen height of 14 mm was established, which was also the largest block size available for production of the CAD/CAM root replicas. The control group of 16 specimens was embedded with polymethyl methacrylate (Paladur; Kulzer GmbH, Hanau, Germany) in sandblasted metal carriers (Figure 1C). Human enamel antagonists were positioned using occlusal paper so as to meet the centric fossa of the crowns. An impression of the embedded specimen’s position and occlusal surface was made with a silicone (Optosil; Kulzer GmbH) and a self-made device, allowing for identical positioning of further specimens (Figure 2).

### 2.4. Adhesive Luting of CAD/CAM Restorations

After milling, the restorations were cleaned in an ultrasonic bath and degreased with ethanol. The luting surface was etched with a 5% hydrofluoric acid (Ceramics Etch; Vita Zahnfabrik, Bad Säckingen, Germany) for 60 s and then rinsed with water for 60 s [40]. Silanization (Monobond Plus; Ivoclar Vivadent, Schaan, Liechtenstein) was performed for 60 s, before a dual-polymerized resin cement (Panavia V5; Kuraray Noritake Dental Inc., Kurashiki, Japan) was used for adhesive luting. The restorations were seated, and finger pressure was applied. After removing excess material, the luting resin cement was polymerized (Bluephase G2; Ivoclar Vivadent, Schaan, Liechtenstein) with 1200 mW/cm^2^ from the occlusal, buccal, lingual, mesial and distal aspect for 40 s each. The preparation of the RC, PM and HC abutments for adhesive luting was performed as proposed by the manufacturer for each material, as presented in Table 2. The prepared teeth were cleaned using an occlubrush (KerrHawe SA, Bioggio, Switzerland) followed by application of a self-etching primer (Panavia V5 Tooth Primer; Kuraray Noritake Dental Inc., Kurashiki, Japan) for 20 s (Table 2).

### 2.5. Thermo-Mechanical Load Cycling and Fracture Loading

After embedment and adhesive cementation, thermo-mechanical load cycling was performed with respect to a standardized protocol in a chewing simulator (CoCom 2, custom-made device at the University of Zurich, Zurich, Switzerland). The protocol was to perform 1.2 million cycles at 1.7 Hz, using an invariable occlusal load of 49 ± 0.7 N, with 12,000 thermal cycles between 5 and 50 °C, a dwell time of 120 s at each temperature, water change time of 10 s and with human molar cusps as the antagonists (Figure 1D) [41]. The specimens were examined for cracks and modes of failures and flawed specimens were eliminated from further testing. This was the case with three specimens of the PM group where cracks were observed. Thereafter, fracture loading was conducted with the intact specimens using a universal testing machine (AllroundLine z010; ZwickRoell, Ulm, Germany). The specimens were firmly attached to the testing machine and a 5.5 mm diameter stainless-steel ball cushioned by a polyethylene film (thickness: 0.1 mm) was placed symmetrically in the central groove of each crown so that the compressive load was evenly applied. With a stainless-steel cylinder mounted on the crosshead of the universal testing machine, compressive force (N) was applied on the steel ball at a crosshead speed of 1 mm/min until fracture. The maximum load at failure was recorded for each specimen. Specimens which failed after thermo-mechanical load cycling were assigned the value 0 N for statistical analysis.

### 2.6. Statistical Analysis

A mixed effect linear model was fitted to the data with fracture load (N) as target variable, group (RC, HD, PM and HC) as the fixed explanatory variable and tooth ID (1–16) as random intercept. Residual analysis was conducted and did not show violations of parametric assumptions (i.e., asymptotic normal distribution and homoscedasticity of residuals). Pairwise contrasts were estimated on the marginal means and corrected for multiple testing according to Tukey. The significance level was set to α = 0.05 and all calculations and graphs were computed using R [42], including the packages ggplot2 [43], lmerTest [44] and emmeans [45].

## 3. Results

**Figure 3:**
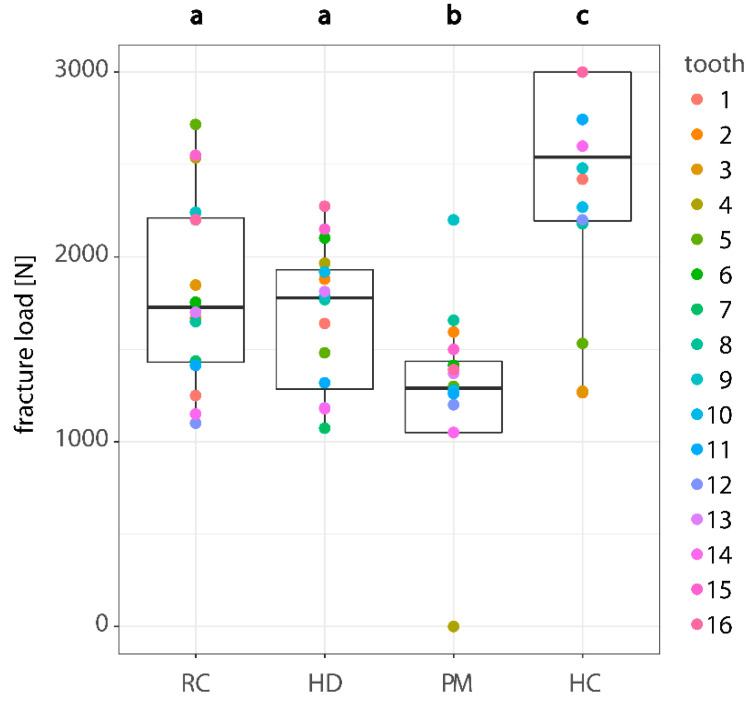
Fracture load (N) according to used abutment material. Dots of same color correspond to specimens with same morphology. Different lowercase letters indicate statistically significant difference of estimated means among abutment materials RC, HD, PM and HC. Medians were 2540 N for HC, 1778 N for HD, 1728 N for RC and 1290 N for PM. RC, reinforced composite; HD, human dentin; PM, polymethyl methacrylate; HC, hybrid ceramic.

The means for fracture load were 2435 N for HC, 1838 N for RC, 1670 N for HD and 1142 N for PM (Table 3). Median was 2540 N for group HC, 1778 N for HD, 1728 N for RC and 1290 N for PM, as visualized in the box plot (Figure 3). The mixed model including Tukey post-hoc analysis revealed significant (*p* < 0.05) differences among all group pairs except for the pair RC and HD (*p* = 0.7676) (Table 3 and Table 4).

PM had three specimens fracture during thermo-mechanical load cycling, which were assigned 0 N. Six specimens of the HC group did not fracture at the maximum compressive force of 3000 N of the universal testing machine; these were assigned the value 3000 N.

## 4. Discussion

The focus of in vitro studies investigating the fracture strength of prosthetic materials has been on the mechanical properties of the reconstruction, rather than the elastic modulus and preparation design of the supporting structure. At best, an abutment material that presumably exhibits a similar elastic modulus as dentin was used for the construction of standardized dies [15]. However, no other study has compared abutment materials with dentin under exact same conditions, in terms of crown and root morphology as well as preparation design.

In this study, CAD/CAM fabricated feldspathic ceramic crowns were luted on prepared human teeth as well as exact replicas thereof made from different CAD/CAM materials. The materials were chosen as having low (PM), medium (RC) and high (HC) elastic modulus. The null hypothesis has to be rejected that no significant differences exist among the fracture resistance of feldspathic ceramic crowns luted on prepared teeth and replicas fabricated from polymethyl methacrylate, reinforced composite and hybrid ceramic.

The mean fracture load for hybrid ceramic (HC) was 2435 N, and 1142 N for polymethyl methacrylate (PM). The human dentin group (HD) exhibited a mean fracture load of 1670 N. These findings agree with previous studies where fracture load values were higher on an abutment material with a higher elastic modulus [10]. Due to the brittle nature of ceramics, higher tensile stress occurs in the crowns during loading on an abutment with a low elastic modulus [12]. Zimmermann et al. investigated the fracture load of feldspathic ceramic crowns on stereolithography fabricated dies with an elastic modulus of 2.5 GPa [46]. The maximum fracture load value of 774 N is similar to the higher values of the PM group (1142 N), where the abutment material’s elastic modulus was 3.2 GPa.

The similarity in fracture load values between the groups HD and RC suggests that in this study the elastic modulus of dentin might be situated in a range around 10.3 GPa. This value is situated at the lower end of the published range of 5–42 GPa for human dentin [4,16,17,18,19,20,21,22,23,24,25,26,27]. A widely used method to measure the elastic modulus of a material is the nanoindentation technique. In this procedure, a diamond indenter of known shape penetrates the surface of a material in the micron or submicron scale [20]. However, it is questionable if elastic modulus values for dentin obtained by similar methods where only a thin surface layer is probed with a very low load are useful to predict the behavior of a prepared tooth with a luted crown when a much greater load is applied. 

The disparity among studies of the elasticity of dentin include its heterogeneous tubular structure, anisotropy and differences in mineralization [19]. Angker et al. described a decrease of the elastic modulus with proximity to the pulp and reported values of around 11.59 GPa near the pulp chamber [17]. This value is compatible with the results of this study and may be attributed to an increase in tubular density and a decrease in the amount of intertubular dentin near the pulp. Therefore, the preparation for a crown may decrease the elastic modulus of the abutment.

Another reason for the differing measured elastic moduli has been reported in the viscoelastic response of dentin. When higher loads, such as physiological strain, are applied over a longer period of time, stress relaxation occurs [18,47]. This process may lead to lower elastic moduli measurements. This viscoelastic behavior leads to lower fracture values than expected. 

In summary, many studies have reported the elastic modulus of dentin to be 20 GPa or higher [19]. However, these studies tested dentin on a micromechanical level, whereas this study examined the behavior of a tooth–crown complex. The presence and size of the pulp chamber might also affect the elastic modulus of the tooth.

In this study, the mean fracture load for reinforced composite (RC) was 1838 N, and 1670 N for dentin (HD), from which we inferred the tooth and composite had similar elastic moduli. Different types of resin composites have been used as dentin analogs [4,8,13,15]. Sagsoz et al. investigated the fracture resistance of feldspathic ceramic crowns on dies fabricated from a Ni-Cr alloy, dentin and an epoxy resin with an elastic modulus of 12.9 GPa. Although the differences among the materials were not significant, the Ni-Cr dies showed the highest mean fracture resistance values (606 N) and the epoxy resin (595 N) exhibited higher values than the dentin dies (578 N), similar to the groups RC and HD [11]. Another study found no statistically significant difference between the fracture strength of zirconia crowns on dies made of dentin or filled epoxy resin having an elastic modulus of 11.8 GPa [48].

The absolute values of these studies are not comparable due to differences in study design, measurement setup and die and crown design. Furthermore, the load which is applied in studies investigating the fracture strength of materials exceeds physiological masticatory loads and can only serve as an approximation to the dynamic clinical situation [49]. For these reasons, the results of these studies must be evaluated in terms of relative values. In this respect, the influence of the die material is visible and leads to statistically significant differences in this specific test setup. Two test groups, showing differences in terms of fracture load with either very high (HC, 30 GPa) and very low (PM, 3.2 GPa) elastic modulus. In contrast, test group RC (10.3 GPa) is located much closer to the reference group of human dentin. From these results, materials with an elastic modulus of around 10–13 GPa may be a viable alternative for in vitro studies investigating mechanical properties of prosthodontic materials.

The study at hand is subject to limitations. Care was taken to position and load the specimens as identically as possible across all groups. However, the fracture pattern still varied among specimens with the same crown morphology. This may be due to the less rigid embedment material of polymethyl methacrylate. Therefore, it was not possible to make a conclusive evaluation of the correlation of the fracture load with regard to tooth shape among the groups. Due to differences in load distribution in the various abutment materials, it is possible that the influence of the morphology on the fracture load diminishes with greater load and the importance of the mechanical properties of the restoration increases.

While the original teeth were scanned and copied to create identical replicas, technical difficulties do exist in accurate reproductions of the roots. The narrow furcation area cannot be reproduced with subtractive CAD/CAM methods, nor can the pulp chamber.

With the existing data, no final recommendation for a specific test group can be drawn as a valid replacement of human dentin dies. There are several factors that need further investigation. The influence of different thermo-mechanical load cycling protocols, of the restorative material and type of cementation have to be evaluated with respect to the underlying die material. In this study, only feldspathic ceramic crowns were used; further studies may be of interest to evaluate the behavior of crowns with higher elastic modulus, such as zirconia.

In the end, only a comparison with the results of in vivo studies may provide the predictability of data from in vitro studies to clinical situations.

## 5. Conclusions

The mechanical properties of the abutment die have an influence on the fracture load of feldspathic crowns. Within the current specific test setup, dies made of composite material are most similar to human dentin.

## Figures and Tables

**Table 1:** Materials.

Commercial Name	Material	Shade	Elastic Modulus (GPa)	Manufacturer
VITABLOCS Mark II	Feldspathic ceramic	3M2C	45	Vita Zahnfabrik Bad Säckingen, Germany
VITA ENAMIC	Hybrid ceramic (HC)	2M2HT	30	Vita Zahnfabrik, Bad Säckingen, Germany
BRILLIANT Crios	Reinforced composite (RC)	A3	10.3	Coltene AG, Altstätten, Switzerland
Telio CAD	Polymethyl methacrylate (PM)	A3	3.2	Ivoclar Vivadent, Schaan, Liechtenstein
-	Human dentin (HD)	-	5–42 [4,16,17,18,19,20,21,22,23,24,25,26,27]	-

Elastic modulus (GPa) values provided by the manufacturers.

**Table 2:** Bonding protocol.

**Preconditioning of Feldspathic Ceramic Crowns**	**Products**
ultrasonic bath 2 min	(Ceramics Etch; Vita Zahnfabrik, Bad Säckingen, Germany) (Monobond Plus; Ivoclar Vivadent, Schaan, Liechtenstein) (Panavia V5; Kuraray Noritake Dental Inc., Kurashiki, Japan) (Bluephase G2; Ivoclar Vivadent, Schaan, Liechtenstein)
ethanol degreasing	
hydrofluoric acid (5%) etching 60 s, rinse 60 s, dry	
silanization 60 s, dry	
application of dual-polymerized resin cement in crown, positioning applying finger pressure, removal of excess	
light polymerization 5 × 40 s (occlusal, buccal, lingual, mesial and distal aspect)	
**Preconditioning of Prepared Human Teeth**	
cleaning	(Occlubrush; KerrHawe SA, Bioggio, Switzerland) (Panavia V5 Tooth Primer; Kuraray Noritake Dental Inc., Kurashiki, Japan)
application of self-etching primer 20 s, dry	
**Preconditioning of Reinforced Composite Dies**	
sandblasting 25–50 μm aluminum oxide, 1.5 bar	(One Coat 7 Universal; Coltene, Altstätten, Switzerland)
ultrasonic bath 2 min	
ethanol degreasing	
application of bond and silanization 20 s, dry	
**Preconditioning of Hybrid Ceramic Dies**	
ultrasonic bath 2 min	(Ceramics Etch; Vita Zahnfabrik, Bad Säckingen, Germany) (Clearfil Ceramic Primer Plus; Kuraray Noritake Dental Inc., Kurashiki, Japan)
ethanol degreasing	
hydrofluoric acid (5%) etching 60 s, rinse 60 s, dry	
application of bond and silanization 20 s, dry	
**Preconditioning of Polymethyl Methacrylate Dies**	
sandblasting 80–100 μm aluminum oxide, 1.5 bar	(SR Connect; Ivoclar Vivadent, Schaan, Liechtenstein) (Bluephase G2; Ivoclar Vivadent, Schaan, Liechtenstein)
ultrasonic bath 2 min	
ethanol degreasing	
silanization 3 min, dry	
light polymerization 40 s	

**Table 3:** Descriptive statistics.

Group	Mean (N)	Median (N)	95% Lower CL (N)	95% Upper CL (N)
RC	1838	1728	1565	2112
HD	1670	1778	1396	1943
PM	1142	1290	868	1415
HC	2435	2540	2162	2709

RC, reinforced composite; HD, human dentin; PM, polymethyl methacrylate; HC, hybrid ceramic.

**Table 4:** Pairwise comparison according to Tukey.

Compared Groups	*p*-Value
RC–HD	0.7676
RC–PM	0.0013 *
RC–HC	0.0069 *
HD–PM	0.0201 *
HD–HC	0.0004 *
PM–HC	<0.0001 *

* Indicates statistically significant difference of estimated means among abutment materials RC, HD, PM and HC; RC, reinforced composite; HD, human dentin; PM, polymethyl methacrylate; HC, hybrid ceramic.

## References

[1] Zhang Y., Kelly J.R. (2017). Dental Ceramics for Restoration and Metal Veneering. Dent. Clin. N. Am..

[2] Land M.F., Hopp C.D. (2010). Survival rates of all-ceramic systems differ by clinical indication and fabrication method. J. Evid. Based Dent. Pract..

[3] Denry I., Holloway J. (2010). Ceramics for Dental Applications: A Review. Materials.

[4] Kelly J.R., Rungruanganunt P., Hunter B., Vailati F. (2010). Development of a clinically validated bulk failure test for ceramic crowns. J. Prosthet. Dent..

[5] Seydler B., Rues S., Müller D., Schmitter M. (2014). In vitro fracture load of monolithic lithium disilicate ceramic molar crowns with different wall thicknesses. Clin. Oral Investig..

[6] Rosentritt M., Steiger D., Behr M., Handel G., Kolbeck C. (2009). Influence of substructure design and spacer settings on the in vitro performance of molar zirconia crowns. J. Dent..

[7] Agustín-Panadero R., Mateos-Palacios R., Román-Rodríguez J.-L., Solá-Ruíz M.-F., Fons-Font A. (2015). Influence of surface preparation on fracture load of resin composite-based repairs. J. Clin. Exp. Dent..

[8] Pallis K., Griggs J.A., Woody R.D., Guillen G.E., Miller A.W. (2004). Fracture resistance of three all-ceramic restorative systems for posterior applications. J. Prosthet. Dent..

[9] Sundh A., Sjögren G. (2006). Fracture resistance of all-ceramic zirconia bridges with differing phase stabilizers and quality of sintering. Dent. Mater..

[10] Scherrer S.S., de Rijk W.G. (1993). The fracture resistance of all-ceramic crowns on supporting structures with different elastic moduli. Int. J. Prosthodont..

[11] Sagsoz N.P., Yanikoğlu N., Sagsoz O. (2016). Effect of Die Materials on the Fracture Resistance of CAD/CAM Monolithic Crown Restorations. Oral Health Dent. Manag..

[12] Anusavice K.J., Tsai Y.L. Stress Distribution in Ceramic Crown Forms as a Function of Thickness, Elastic Modulus, and Supporting Substrate. Proceedings of the 16th Southern Biomedical Engineering Conference.

[13] Bindl A., Lüthy H., Mörmann W.H. (2006). Strength and fracture pattern of monolithic CAD/CAM-generated posterior crowns. Dent. Mater..

[14] Peutzfeldt A., Sahafi A., Flury S. (2011). Bonding of restorative materials to dentin with various luting agents. Oper. Dent..

[15] Nakamura K., Harada A., Inagaki R., Kanno T., Niwano Y., Milleding P., Örtengren U. (2015). Fracture resistance of monolithic zirconia molar crowns with reduced thickness. Acta Odontol. Scand..

[16] Kinney J.H., Balooch M., Marshall G.W., Marshall S.J. (1999). A micromechanics model of the elastic properties of human dentine. Arch. Oral Biol..

[17] Angker L., Swain M.V., Kilpatrick N. (2003). Micro-mechanical characterisation of the properties of primary tooth dentine. J. Dent..

[18] Kinney J.H., Marshall S.J., Marshall G.W. (2003). The mechanical properties of human dentin: A critical review and re-evaluation of the dental literature. Crit. Rev. Oral Biol. Med..

[19] Zhang Y.-R., Du W., Zhou X.-D., Yu H.-Y. (2014). Review of research on the mechanical properties of the human tooth. Int. J. Oral Sci..

[20] Ziskind D., Hasday M., Cohen S.R., Wagner H.D. (2011). Young’s modulus of peritubular and intertubular human dentin by nano-indentation tests. J. Struct. Biol..

[21] Kinney J.H., Balooch M., Marshall S.J., Marshall G.W., Weihs T.P. (1996). Hardness and Young’s modulus of human peritubular and intertubular dentine. Arch. Oral Biol..

[22] Sano H., Ciucchi B., Matthews W.G., Pashley D.H. (1994). Tensile properties of mineralized and demineralized human and bovine dentin. J. Dent. Res..

[23] Meredith N., Sherriff M., Setchell D.J., Swanson S.A. (1996). Measurement of the microhardness and Young’s modulus of human enamel and dentine using an indentation technique. Arch. Oral Biol..

[24] Habelitz S., Marshall G.W., Balooch M., Marshall S.J. (2002). Nanoindentation and storage of teeth. J. Biomech..

[25] Marshall G.W., Habelitz S., Gallagher R., Balooch M., Balooch G., Marshall S.J. (2001). Nanomechanical properties of hydrated carious human dentin. J. Dent. Res..

[26] Mahoney E., Holt A., Swain M., Kilpatrick N. (2000). The hardness and modulus of elasticity of primary molar teeth: An ultra-micro-indentation study. J. Dent..

[27] Marshall G.W., Marshall S.J., Kinney J.H., Balooch M. (1997). The dentin substrate: Structure and properties related to bonding. J. Dent..

[28] Balooch M., Wu-Magidi I.C., Balazs A., Lundkvist A.S., Marshall S.J., Marshall G.W., Siekhaus W.J., Kinney J.H. (1998). Viscoelastic properties of demineralized human dentin measured in water with atomic force microscope (AFM)-based indentation. J. Biomed. Mater. Res..

[29] Goldberg M., Kulkarni A.B., Young M., Boskey A. (2011). Dentin: Structure, Composition and Mineralization: The role of dentin ECM in dentin formation and mineralization. Front. Biosci. (Elite Ed).

[30] Ruse N.D., Sadoun M.J. (2014). Resin-composite blocks for dental CAD/CAM applications. J. Dent. Res..

[31] Manicone P.F., Rossi Iommetti P., Raffaelli L. (2007). An overview of zirconia ceramics: Basic properties and clinical applications. J. Dent..

[32] Stawarczyk B., Eichberger M., Hoffmann R., Noack F., Schweiger J., Edelhoff D., Beuer F. (2014). A novel CAD/CAM base metal compared to conventional CoCrMo alloys: An in-vitro study of the long-term metal-ceramic bond strength. Oral Health Dent. Manag..

[33] Fernández-Estevan L., Millan-Martínez D., Fons-Font A., Agustín-Panadero R., Román-Rodríguez J.-L. (2017). Methodology in specimen fabrication for in vitro dental studies: Standardization of extracted tooth preparation. J. Clin. Exp. Dent..

[34] Colombo M., Poggio C., Lasagna A., Chiesa M., Scribante A. (2019). Vickers Micro-Hardness of New Restorative CAD/CAM Dental Materials: Evaluation and Comparison after Exposure to Acidic Drink. Materials.

[35] Jang Y.S., Oh S.H., Oh W.S., Lee M.H., Lee J.J., Bae T.S. (2019). Effects of Liner-Bonding of Implant-Supported Glass-Ceramic Crown to Zirconia Abutment on Bond Strength and Fracture Resistance. Materials.

[36] Yin R., Jang Y.S., Lee M.H., Bae T.S. (2019). Comparative Evaluation of Mechanical Properties and Wear Ability of Five CAD/CAM Dental Blocks. Materials.

[37] Aydın B., Pamir T., Baltaci A., Orman M.N., Turk T. (2015). Effect of storage solutions on microhardness of crown enamel and dentin. Eur. J. Dent..

[38] Goodacre C.J., Campagni W.V., Aquilino S.A. (2001). Tooth preparations for complete crowns: An art form based on scientific principles. J. Prosthet. Dent..

[39] Otto T., Mörmann W.H. (2015). Clinical performance of chairside CAD/CAM feldspathic ceramic posterior shoulder crowns and endocrowns up to 12 years. Int. J. Comput. Dent..

[40] Venturini A.B., Prochnow C., Rambo D., Gundel A., Valandro L.F. (2015). Effect of Hydrofluoric Acid Concentration on Resin Adhesion to a Feldspathic Ceramic. J Adhes Dent..

[41] Krejci I., Reich T., Lutz F., Albertoni M. (1990). In-vitro-Testverfahren zur Evaluation Dentaler Restaurationssysteme. 1. Computergesteuerter Kausimulator. Schweiz. Monatsschr. Zahnmed..

[42] RCore Team R: A Language and Environment for Statistical Computing. https://www.R-project.org/.

[43] Wickham H. (2016). ggplot2. Elegant Graphics for Data Analysis.

[44] Kuznetsova A., Brockhoff P.B., Christensen R.H.B. (2017). lmerTest Package: Tests in Linear Mixed Effects Models. J. Stat. Soft..

[45] Lenth R. emmeans: Estimated Marginal Means, Aka Least-Squares Means. https://CRAN.R-project.org/package=emmeans.

[46] Zimmermann M., Egli G., Zaruba M., Mehl A. (2017). Influence of material thickness on fractural strength of CAD/CAM fabricated ceramic crowns. Dent. Mater. J..

[47] Korostoff E., Pollack S.R., Duncanson M.G. (1975). Viscoelastic properties of human dentin. J. Biomed. Mater. Res..

[48] Yucel M.T., Yondem I., Aykent F., Eraslan O. (2012). Influence of the supporting die structures on the fracture strength of all-ceramic materials. Clin. Oral Investig..

[49] Morneburg T.R., Proschel P.A. (2002). Measurement of masticatory forces and implant loads: A methodologic clinical study. Int. J. Prosthodont..

